# Satisfaction with health care services in young people with cerebral palsy in the transition period: results from a European multicenter study

**DOI:** 10.3389/fmed.2024.1306504

**Published:** 2024-01-30

**Authors:** Holger Muehlan, Joaquim Alvarelhao, Catherine Arnaud, Chirine Cytera, Jerome Fauconnier, Kate Himmelmann, Marco Marcelli, Henriette Markwart, Marion Rapp, Silke Schmidt, Ute Thyen

**Affiliations:** ^1^Department Health and Prevention, University of Greifswald, Greifswald, Germany; ^2^School of Health Sciences, University of Aveiro, Aveiro, Portugal; ^3^UMR 1295 CERPOP Centre for Epidemiology and Research in POPulation Health, Inserm, Université Toulouse III - Paul Sabatier, Team SPHERE, Toulouse, France; ^4^Clinical Epidemiology Unit, University Hospital, Toulouse, France; ^5^Hospital for Child and Adolescent Medicine, University of Luebeck, Luebeck, Germany; ^6^Laboratoire TIMC-IMAG Equipe ThEMAS, Pavillon Taillefer, Université Joseph Fournier, Grenoble, France; ^7^Department of Pediatrics, Sahlgrenska Academy, University of Gothenburg, Gothenburg, Sweden; ^8^Azienda Sanitaria Locale Viterbo, Child and Adolescent Neuropsychiatric Unit—Adult Disability Unit, Viterbo, Italy; ^9^Institute for Community Medicine, University Medicine Greifswald, Greifswald, Germany

**Keywords:** cerebral palsy, chronic condition, emerging adulthood, young adults, transition, special needs, healthcare utilization, satisfaction with care

## Abstract

**Background:**

Young people with chronic health conditions and disabilities rely on the healthcare system to maintain their best possible health. The appropriate delivery and utilization of healthcare services are key to improve their autonomy, self-efficacy and employment outcomes. The research question of our study is directed toward investigating if poor availability and accessibility of healthcare services in general, as identified by unmet needs in healthcare, are associated with dissatisfaction with healthcare.

**Methods:**

Within a European multicenter observational study, 357 young adults with cerebral palsy aged 19–28 were included. We assessed special healthcare needs, utilization of healthcare services, and satisfaction with healthcare applying the short-form of the YHC-SUN-SF, environmental and social variables (EAEQ) as well as indicators for severity of condition and functionality (e.g., GMFCS) of these participants based on a self-, assisted self- or proxy-reports. We used correlation analyses to explore associations between satisfaction with healthcare and respective indicators related to availability and accessibility of healthcare services as well as severity of the condition. In addition, we included reference values for satisfaction with heath care from young adults with various chronic conditions assessed within population-based surveys from some of the European countries included in the study.

**Results:**

We identified several unmet healthcare needs, especially for widely used and established services (e.g., physical therapy). Satisfaction with healthcare (YHC-SUN-SF general and subscale scores) was moderate to high and almost consistently better for the sample of young adults with cerebral palsy as compared to reference values for young adults with various chronic conditions assessed within general population surveys). Correlation coefficients between satisfaction with healthcare and utilization of services and (unmet) healthcare needs were low, also with different indicators for severity of the condition or functionality.

**Conclusion:**

Young adults with cerebral palsy reports of unmet healthcare needs varied largely but showed substantial deficits in some aspects. This seems to have no impact on the satisfaction with healthcare those patients currently receive. We conclude that these are two different constructs and somewhat independent indicators to evaluate the quality of healthcare. Clinicians and other practitioners should consider this distinction when monitoring patient needs in their daily practice.

## Introduction

Over the last two decades, birth prevalence for cerebral palsy (CP) caused in the prenatal or perinatal period decreased substantially to 1.5 per 1000 live births in high income countries, i.e., Europe and Australia, while in regions of the global south it remained or increased to 3.4 per 1000 live births (1, 2). The reasons for the decline in more affluent regions are not fully understood, but better healthcare may play a role (3). Thus, access to healthcare services play an important role in preventing CP. However, the course of the condition and functional outcomes also depends on good quality healthcare. The impact of this lifelong condition often associated with severe disability makes CP a significant condition from a public health perspective (4). People with chronic health conditions and disabilities rely on the healthcare system to maintain their best possible health. Generally, they require interdisciplinary services within the healthcare system and multisectoral cooperation with social services, employment, housing, education and community resources for communication and mobility. Healthcare services can play an important part to improve, e.g., autonomy, self-efficacy, and employment outcomes (5).

The transitioning from pediatric care to adult medicine is associated with challenges for young people with special healthcare needs (6). This is accompanied by risks of a deterioration of care, the underutilization of services and ultimately poor health outcomes. Gaps in transitional care had been identified and recommendations for best practice were established (7, 8). Also, for young patients with widespread chronic diseases, care structures to address the obstacles associated with the transition process were established, e.g., transition programs (9). Such programs have also been introduced for young people with CP (10), but their availability and accessibility is far from being comprehensive, so does the fulfillment of the care needs of these patients, which are diversified and special (11, 12). The transition process must be individualized to the developmental needs of the adolescent/young adult and gradually work toward more autonomy. A recent qualitative study reported that the transition to adulthood for young adults with CP was far from gradual and was perceived as being “thrust into adulthood” (13).

Our research question is directed toward investigating if poor availability and accessibility of healthcare services are associated with dissatisfaction with healthcare. Given that limited access is known to be associated with impaired healthcare satisfaction, we assume that poor access to healthcare services, identified by unmet needs in healthcare, is associated with dissatisfaction with healthcare. To account for the patient’s perspective, we use satisfaction with healthcare as a surrogate marker for good and adequate healthcare provision, given that patient-reported experience measures have been widely established in interdisciplinary healthcare and implementation studies.

## Materials and methods

### Design and samples

#### Cerebral palsy sample (CP)

We used data from SPARCLE3, a multicenter European observational population-based study combining the follow-up of the SPARCLE cohort to young adulthood (19–28 years) and a cross-sectional part allowing the recruitment of a larger sample of young people with CP. SPARCLE3 was implemented in five of the nine European regions originally investigated: South West and South East France (Haute-Garonne and Isère counties, respectively), North West Germany, Western Sweden (region of Goteborg), Central Italy (Viterbo area). The region of Porto (Portugal) participated in the cross-sectional part. Further information is provided in the study protocol (14). The SPARCLE3 sample included young adults born between 31/07/1991 and 01/04/1997 with confirmed diagnosis of CP as defined by the SCPE (15). Out of 357 participants, 110 came from Germany, 105 from Portugal, 88 from France, 30 from Sweden, and 24 from Italy.

#### General population sample (GP)

Both in France and Germany, a population-based comparison group of young adults of the same age group was recruited with young adults participating in an online survey. Participants of the survey received incentives by a panelist who recruited males and females (in 50%/50% ratio) aged 19–29 years living in one of both participating countries (*N* = 4.051; France: *n* = 2080; Germany: *n* = 1964). In addition, comparison data from the other countries were available as well, but not on a population-wide level (Sweden: *n* = 987; Portugal: *n* = 105; Italy: *n* = 24).

### Instruments

#### Youth Health Care - Satisfaction, Utilization & Needs - Short-Form measure (YHC-SUN-SF)

Specifically developed for adolescent self-report (age 15–25) on their satisfaction with their care provision (16), the YHC-SUN was derived from the CHC-SUN, reported by parents (17). This version was shortened to 30 items in SPARCLE3. The first module “*Receipt of services*” comprises 7 items plus the list of unmet needs (16 items) and the second module “*Satisfaction with care*” comprises 7 items (18). Answering options included: (a) services both needed and fully or partly used, (b) services not needed and not used, and (c) services needed but not used. We labeled the latter category as “unmet need” from the perspective of the user. Written in German, this questionnaire had no official translation available. The SPARCLE3 version has been translated with forward-backward procedure from German to English (in Lübeck) and afterwards by every country on their own in their language. Three following subscale-scores were calculated: “diagnosis/information,” “doctors’ behavior,” and “patient centered care.” The domains represented by these subscales were originally chosen for the short-form measure derived from the model of the original long-form measure, because all relate to the interaction between physician and patient. Item content covers satisfaction with information given about the condition and treatment choices, about the way doctors listened and explained as well about time spent and efforts made for the consultation. The YHC-SUN-SF measure was applied in both samples (CP, GP), except for the GP subsample from France.

#### European Adolescent Environment Questionnaire (EAEQ)

This instrument was developed in the SPARCLE project as an adaptation of the ECEQ [European Child Environment Questionnaire, (19)]. Lindsay Pennington (Newcastle) and Joaquim Alvarelhao (Porto) developed a new version specific for adulthood, comprising 61 items related to the physical, social and attitudinal environments. The questionnaire is divided into 8 parts: Physical environment at home (6 items), Physical environment at work/university/in day placement (5 items), Physical environment in public places (6 items), Access to transport (7 items), Access to health services and carer (9 items), Financial support (5 items), Attitude and support (20 items), Access to information (4 items). For this paper we used items from the Dimension “Access to health services and carer.” The question “Do you need…?” offers the answer categories “no” and “yes,” if yes a question follows whether the service is available or not. The EAEQ was assessed in the CP sample only.

#### Severity assessment

We also collected data on gross motor function (GMFCS) (20–22), hand function [Bimanual Fine Motor Function (BFMF)] (23), communication [Functional Communication Classification System (FCCS)] (24), and feeding (Eating and Drinking Ability Classification System (EDACS) (25), and vision and hearing impairments. Cognitive level was estimated by combining: information from the CP register at age five or later years, neuropsychological assessment if available, current school performance, and ability to self-report. Seizure frequency in the last year and the use of anticonvulsants were recorded. Severity assessments were applied in the CP sample only.

#### General health

The standard question “How is your health in general? Would you say it is …” was used in the European Social Survey (ESS) from round 1 (2002) to round 9 (2018). Respondents were asked to rate their current health on a five-step ladder ranging from very bad (1) to very good (5). Translations exist for SPARCLE countries (France, Germany, Italy, Portugal, Sweden). In 2016, 66.7% of the EU-28 population aged 18 and over reported that their health status was good or very good. At the other end of the spectrum, almost 1 in 10 (9.1%) persons perceived their health status to be bad or very bad (26). General health was assessed in both samples (CP, GP).

### Data analysis

Descriptive statistics were used to describe the respondents’ socio-demographic as well as clinical characteristics. Statistical tests (ANOVA/χ^2^-test) as well as corresponding effect size measures and *p*-values were calculated to provide indicators of group differences. Spearman correlation analysis was used to investigate statistical associations between YHC-SUN-SF scores for satisfaction with care and different indicators for severity of condition and functioning. All quantitative statistical analyses were conducted using IBM SPSS Statistics 28.0.

## Results

### Sample

Among the 357 participants from the CP sample, 157 were female (44%), 216 lived with parents (60.5%), about 10% in facilities and 18% independently (Table 1A). A total of 117 responded by proxy-report by a carer (32.8%), less than a fifth had achieved more than secondary education. A total of 319 (89.4%) had a general practitioner and 236 (66.1%) a specialist for the condition. Young adults with CP had a mean age of 24.0 years (SD = 1.6 years) at the time of interview. Educational attainment was much lower in the CP group. Noteworthy, self-rated health status (range 1–5) does not differ between the CP sample (*M* = 2.08, SD = 0.82) and the GP sample (*M* = 2.08, SD = 0.81; *p* = 0.40, η = 0.01).

**TABLE 1A:** Characteristics of both samples.

		Cerebral palsy (CP)		General population (GP)		ANOVA/χ ^2^-test
		*N*	%	*N*	%	
Country						χ^2^(1,4) = 515.87, *p* < 0.001
	France	88	24.6%	2080	40.8	
	Germany	110	30.8%	1964	38.5	
	Italy	24	6.7%	24	0.5	
	Portugal	105	29.4%	47	0.9	
	Sweden	30	8.4%	987	19.3	
Sex						χ^2^(1,1) = 9.60, *p* < 0.002
	Male	200	56.0%	2426	47.5%	
	Female	157	44.0%	2676	52.5%	
Age						*F*_(1,5457)_ = 5.66, *p* < 0.02, η = 0.03
	*Mean (SD), years*	*24.03*	*(2.01)*	*24.45*	*(3.29)*	
Living environment						χ^2^(1,4) = 16.50, *p* = 0.002
	A big city (population over 200,000)	96	26.9%	1629	31.9%	
	The suburbs or outskirts of a big city (population over 200,000)	30	8.4%	668	45.0%	
	A town or a small city (population between 3,000 and 200,000)	159	44.5%	2070	40.6%	
	A country village (population less than 3,000)	61	17.1%	621	12.2%	
	A farm or home in the country	9	2.5%	113	2.2%	
	*(Missing)*	2	0.6%	1	0%	
Education						χ^2^(1,8) = 578.65, *p* = 0.001
	Early childhood educational development	111	31.1%	91	1.8%	
	Primary education	33	9.2%	45	0.9%	
	Lower secondary education	65	18.2%	559	11.0%	
	Upper secondary education	83	23.2%	1448	28.4%	
	Post-secondary non-tertiary education	12	3.4%	745	14.6%	
	Short cycle tertiary education	11	3.1%	612	12.0%	
	Bachelor or equivalent level	21	5.9%	927	18.2%	
	Master or equivalent level	16	4.5%	611	12.0%	
	Doctoral or equivalent level	–	–	61	1.2%	
	*(Missing)*	5	1.4%	3	0.1%	
Years of education						*F*_(1,5405)_ = 26.06, *p* = 0.001, η = 0.07
	*Mean (SD)*	*11.75*	*(4.95)*	*13.14*	*4.93*	
Type of report						*(n.a.)*
	Self-report without assistance	134	37.5%	5102	100%	
	Self-report with assistance	105	29.4%	–	–	
	Proxy-report	117	32.8%	–	–	
	*(Missing)*	1	0.3%	–	–	
**General health**						
	*Mean (SD)*	*2.08*	*(0.82)*	*2.04*	*0.81*	*F*_(1,5456)_ = 0.71, *p* = 0.40, η = 0.01
	Very good	84	23.5%	1267	24.8%	χ^2^(1,4) = 1.66, *p* = 0.80
	Good	181	50.7%	2618	51.3	
	Fair	75	21.0%	978	19.2%	
	Bad	14	3.9%	214	4.2%	
	Very bad	3	0.8%	24	0.5%	
	*(Missing)*	–	–	1	0%	

Subtypes of CP represented in the sample were spastic unilateral (26.3%), spastic bilateral (54.9%), dyskinetic (10.5%) and ataxic (7.0%). Gross motor function (GMFCS) indicates that for nearly 40% of the participants with CP moving was either limited (18.2%) or even severely limited (20.2%), whereas more than one third is able to walk and climb stairs (35.3%). Around two third are able to communicate effectively in most situations on their own (58.8%) or with some help (7.6%), as indicated by the BFMFS. Further clinical characteristics may be found in Table 1B.

**TABLE 1B:** Clinical and functional characteristics of sample with young adults with cerebral palsy (CP sample, *n* = 357).

Type of CP		*N*	%
	Spastic unilateral	94	26.3%
	Spastic bilateral	196	54.9%
	Dyskinetic	35	9.8%
	Dyskinetic chorea-athetotic	6	1.7%
	Ataxic	25	7.0%
	*(Missing)*	1	0.3%
**GMFCS**	*Mean (SD)*	*2.74*	*1.58*
I	Child walks and climbs stairs	126	35.3%
II	Child walks inside	50	14.0%
III	Child walks with limitations	44	12.3%
IV	Moving about is limited	65	18.2%
V	Moving about is severely limited	72	20.2%
**BFMF**	*Mean (SD)*	*2.58*	*1.43*
I	Without limitation	114	31.9%
II	Both hands limited in fine skills	80	22.4%
III	Child needs help with tasks	55	15.4%
IV	Child needs help and adapted equipment	59	16.5%
V	Child needs total human assistance	49	13.7%
**FCCS**	*Mean (SD)*	*2.12*	*1.50*
I	Effective communicator in most situations	210	58.8%
II	Effective communicator in most situations, but does need some help	27	7.6%
III	Effective communicator in some situations, small range of messages/topics to most familiar people	26	7.3%
IV	Assistance required in most situations, especially with unfamiliar people and communicates daily/routine needs and wants	56	15.7%
V	Communicates unintentionally with others, using movements and behavior	38	10.6%
**EDACS**	*Mean (SD)*	*1.84*	*1.24*
I	Safely and efficiently	215	60.2%
II	Safely but with some limitations to efficiency	51	14.3%
III	With some limitations to safety, maybe limitations to efficiency	45	12.6%
IV	With significant limitations to safety	24	6.7%
V	Unable to eat or drink safely/tube feeding	22	6.2%

CP, cerebral palsy; GMFCS, gross motor function classification system; BFMF, bimanual fine motor function; FCCS, functional communication classification system; EDACS, eating and drinking ability classification system; SD, standard deviation.

### Unmet healthcare needs

We found a substantial number of unmet healthcare needs (Table 2): Subjectively reported unmet healthcare needs exceeded 10% (up to 17%) in most areas (12 out of 16, 75%), except for physical aids (5%) and communication aids (6%), home nursing (6%) as well as nutrition counseling (8%). Noteworthy, from those who reported not to get physiotherapy (33%, *n* = 118), almost a half stated they would need it (47%, *n* = 55); among those without support with filling applications (58%, *n* = 208), approximately a quarter would need it (28%, *n* = 58), a similar proportion appears (24%, *n* = 61) among those who reported not to get occupational therapy (69%; *n* = 245).

**TABLE 2:** Healthcare needs as assessed with the YHC-SUN-SF (CP sample, *n* = 357).

Do you get…	Missing		Yes		Yes, partly		No and no need		No, but need		No receipt (total)	
	*n*	*%*	*n*	*%*	*n*	*%*	*n*	*%*	*n*	*%*	*n*	*%*
Physiotherapy	0	*0.0*	211	*58.9*	29	*8.1*	62	*17.3*	55	*15.4*	117	*33.0*
Physical aids	1	*0.3*	172	*48.2*	54	*15.1*	113	*31.7*	17	*4.8*	130	*36.4*
Support with filling applications	1	*0.3*	116	*32.5*	32	*9.0*	150	*42.0*	58	*16.2*	208	*58.3*
Occupational therapy	1	*0.3*	83	*23.2*	28	*7.8*	184	*51.5*	61	*17.1*	245	*68.6*
Social services	0	*0.0*	77	*21.5*	31	*8.7*	196	*54.7*	53	*14.8*	249	*69.6*
Health services at work or school	5	*1.4*	63	*17.8*	25	*7.1*	228	*64.6*	36	*10.2*	264	*74.8*
Psychotherapy	2	*0.6*	68	*19.1*	16	*4.5*	213	*59.8*	58	*16.3*	271	*76.1*
Nutrition counseling	1	*0.3*	63	*17.6*	9	*2.5*	256	*71.7*	28	*7.8*	284	*79.6*
Speech therapy	0	*0.0*	47	*13.1*	12	*3.4*	245	*68.4*	53	*14.8*	298	*83.2*
Information	2	*0.6*	32	*9.0*	23	*6.5*	242	*68.0*	58	*16.3*	300	*84.3*
Communication aids	2	*0.6*	36	*10.1*	19	*5.3*	279	*78.2*	22	*6.2*	301	*84.3*
Home nursing	1	*0.3*	41	*11.5*	11	*3.1*	283	*79.3*	21	*5.9*	304	*85.2*
Rehabilitation	1	*0.3*	58	*16.2*	14	*3.9*	255	*71.4*	59	*16.5*	314	*88.0*
Self-help group	2	*0.6*	19	*5.3*	9	*2.5*	273	*76.7*	54	*15.2*	327	*91.9*
Telephone advice by medical professional	0	*0.0*	27	*7.5*	18	*5.0*	292	*81.6*	40	*11.2*	332	*92.7*
Counseling about sexuality	3	*0.8*	7	*2.0*	15	*4.2*	292	*82.3*	40	*11.3*	332	*93.5*

Special healthcare needs were assessed using two different assessments (YHC-SUN-SF, EAEQ). The overlap between corresponding responses of both measures is printed in bold in Table 3, ranging between 80 and 98% for physical therapy, occupational therapy and speech therapy. For the assessment of healthcare needs of other services, the terms used vary between both assessments and the range of corresponding responses is substantially impaired (self-help/support groups: 63–82%; social/counseling services: 43–70%) as compared to the aforementioned services.

**TABLE 3:** Overlap of corresponding responses to YHC-SUN-SF and EAEQ items (CP sample, *n* = 357).

YHC-SUN-SF question: “Do you get…”*		EAEQ question: “Do you need…”*					
		…Physiotherapy?					
	Total**	No		Yes, I have it		Yes, but I don’t have it	
	*N*	*n*	*%*	*n*	*%*	*n*	*%*
Yes, fully or partly	240	2	*0.8*	**228**	***95.0***	10	*4.2*
No, not needed	62	**58**	***93.5***	2	*3.2*	2	*3.2*
No, but I need it	54	4	*7.4*	4	*7.4*	**46**	***85.2***
(Missing)***	1						
		**…Occupational therapy?**					
		**No**		**Yes, I have it**		**Yes, but I don’t have it**	
		***n***	***%***	***n***	***%***	***n***	***%***
Yes, fully or partly	110	11	*10.0*	**95**	***86.4***	4	*3.6*
No, not needed	184	**172**	***93.5***	5	*2.7*	7	*3.8*
No, but I need it	60	9	*15.0*	1	*1.7*	**50**	***83.3***
(Missing)	3						
		**…Speech therapy?**					
		**No**		**Yes, I have it**		**Yes, but I don’t have it**	
		***n***	***%***	***n***	***%***	***n***	***%***
Yes, fully or partly	59	6	*10.2*	**47**	***79.7***	6	*10.2*
No, not needed	245	**239**	***97.6***	0	*0.0*	6	*2.4*
No, but I need it	52	8	*15.4*	0	*0.0*	**44**	***84.6***
(Missing)	1						
**Do you have access to self-help groups?**		**Do you need support groups in your area?**					
		**No**		**Yes, I have it**		**Yes, but I don’t have it**	
		***n***	***%***	***n***	***%***	***n***	***%***
Yes, fully or partly	28	5	*17.9*	**20**	***71.4***	3	*10.7*
No, not needed	271	**223**	***82.3***	20	*7.4*	28	*10.3*
No, but I need it	54	16	*29.6*	4	*7.4*	**34**	***63.0***
(Missing)	4						
**Do get advice from social services?**		**Do you need counseling services?**					
		**No**		**Yes, I have it**		**Yes, but I don’t have it**	
		***n***	***%***	***n***	***%***	***n***	***%***
Yes, partly	107	44	*41.1*	**46**	***43.0***	16	*15.0*
No, not needed	196	**138**	***70.4***	39	*19.9*	19	*9.7*
No, but I need it	53	16	*30.2*	10	*18.9*	**27**	***50.9***
(Missing)	1						

*Corresponding assessments of both questions are printed in bold.

**Percentages are calculated based on row-wise frequency of cases.

***Number of missing values varies for each pair of variables (YHC-SUN-SF, EAEQ). EAEQ, European Adolescent Environment Questionnaire; YHC-SUN-SF, Youth Health Care - Satisfaction, Utilization and Needs - Short Form.

### Satisfaction with care

YHC-SUN-SF total and subscale (unweighted mean) scores as well as the single-item measure score (range 1–5) indicated moderate to high levels of satisfaction for the three different dimensions of healthcare with scores almost consistently higher on a significant level for the sample of young adults with CP as compared with data of young adults from the general population (Table 4).

**TABLE 4:** Satisfaction with care (YHC-SUN-SF) for young adults with cerebral palsy from the SPARCLE study (*n* = 357) and young adults with different types of chronic health conditions from the general population in different European countries (GP sample, *n* = 14–703).

YHC-SUN-SF scores			Subscale “Information/ Diagnosis”	Subscale “Behavior of the doctor”	Subscale “Patient centered care”	Short-Form Global Score	Satisfaction with healthcare (single item)
		*N*	M (SD)	M (SD)	M (SD)	M (SD)	M (SD)
Cerebral palsy	France	88	3.22 (0.93)	3.69 (1.00)	3.57 (1.07)	3.45 (0.85)	3.35 (0.91)
	Germany	110	2.90 (1.01)	3.65 (1.05)	3.46 (1.01)	3.32 (0.87)	3.35 (1.02)
	Italy	24	3.00 (0.96)	3.52 (1.13)	3.39 (1.14)	3.24 (0.97)	2.79 (0.93)
	Portugal	105	3.37 (0.96)	3.57 (0.97)	3.42 (1.02)	3.45 (0.87)	3.34 (0.99)
	Sweden	30	2.76 (1.08)	3.52 (1.07)	3.22 (1.11)	3.12 (0.99)	3.07 (1.02)
	*Total (ANOVA)*	*345*	*F*_(1,4)_ = 4.21, *p* = 0.002, η = *0.05*	*F*_(1,4)_ = 0.33, *p* = 0.86, η = 0.01	*F*_(1,4)_ = 0.65, *p* = 0.63, η = 0.01	*F*_(1,4)_ = 1.14, *p* = 0.0.35, η = *0.01*	*F*_(1,4)_ = 2.16, *p* = 0.07, η = 0.02
General population*	France**	–	–	–	–	–	–
	Germany	703	2.82 (1.03)	2.98 (1.08)	3.11 (1.03)	2.96 (0.92)	3.04 (1.06)
	Italy ***	–	–	–	–	–	–
	Portugal	14	2.61 (1.06)	3.43 (1.12)	3.68 (1.14)	3.24 (0.91)	3.14 (1.10)
	Sweden	217	2.37 (1.02)	2.70 (1.21)	2.88 (1.22)	2.64 (0.98)	2.57 (1.09)
	*Total (ANOVA)*	*935*	*F*_(1,3)_ = 10.69, *p* = 0.000, η = 0.03	*F*_(1,3)_ = 4.57, *p* = 0.003, η = 0.02	*F*_(1,3)_ = 4.24, *p* = 0.005, η = 0.01	*F*_(1,3)_ = 7.22, *p* = 0.000, η = 0.02	*F*_(1,3)_ = 11.57, *p* = 0.000, η = 0.04
ANOVA between both samples			*F*_(1,1272)_ = 38.88, *p* = 0.00, η = 0.03	*F*_(1,1279)_ = 102.84, *p* = 0.00, η = 0.07	*F*_(1,1276)_ = 34.26, *p* = 0.00, η = 0.03	*F*_(1,1279)_ = 67.77, *p* = 0.00, η = 0.05	*F*_(1,1290)_ = 29.46, *p* = 0.00, η = 0.02

*Subsamples of the general population surveys including a selection of young adults with chronic health conditions only.

**The YHC-SUN-SF measure was not applied in the general population survey in France.

***Data from Italy are not reported, the subsample with a chronic condition is too small (*n* = 2). M, mean score; SD, standard deviation; YHC-SUN-SF, Youth Health Care - Satisfaction, Utilization and Needs - Short Form.

### Associations between satisfaction with care and unmet needs and severity of condition

Associations between satisfaction with care and the status of the chronic health condition in the CP sample: With a few exceptions, there were very low negative associations between healthcare satisfaction (YHC-SUN-SF) with different indicators of severity of the condition and functioning, respective correlations coefficients ranging from *r* = −0.17 to *r* = 0.08 (Table 5).

**TABLE 5:** Correlation coefficients for association between YHC-SUN-SF scores for satisfaction with care and different indicators for severity of condition and functioning (CP sample, *n* = 357).

Satisfaction with healthcare (YHC-SUN-SF)	*GMFCS*	*BFMF*	*EDACS*	*FCCS*	*Visual impairment*	*Hearing impairment*	*Seizures*	*Speaking Ability*
Global score	−0.126*	−0.148**	−0.112	−0.156*	−0.116	0.054	−0.009	−0.115*
”Information/ Diagnosis”	−0.147*	−0.173**	−0.112	−0.128*	−0.100	0.047	−0.022	−0.092
“Behavior of the doctor”	−0.082	−0.117*	−0.078	−0.112*	−0.086	0.039	0.004	−0.090
“Patient centered care”	−0.093	−0.103*	−0.097	−0.133*	−0.053	0.079	0.029	−0.101*

Spearman correlation coefficients.

*p ≤ 0.05;

**p ≤ 0.01.

Moreover, except for provision of physical aids, rehabilitation measures, and nutritional/counseling therapy, low correlations coefficients were detected between satisfaction with care (YHC-SUN-SF) and utilization of healthcare services or healthcare needs for 3 of the 16 services assessed by the first module of the YHC-SUN-SF module (for a comprehensive list of these services see Table 3).

## Discussion

Our study explored unmet needs in healthcare in young adults with CP and how these are associated with dissatisfaction with healthcare. We assumed that poor access to healthcare, identified by unmet needs in healthcare, is associated with dissatisfaction with healthcare.

We found a substantial number of *unmet healthcare needs*, especially for widely used and established services such as physical therapy and occupational therapy. One third of participants reported that they did not receive physical therapy; 17.3% stated that they did not perceive the need for such service and 15.4% reported unmet needs. Two thirds of the participants did not receive occupational therapy, 42% of respondents perceived no need for such services; however, the rate of unmet needs was high with 17.1%. This result confirms results provided by a recent study from Ireland (27). The authors report similar rates of unmet needs in physical therapy (23%) and occupational therapy (13%). A study with a convenience sample recruited online in France reported that finding an available physiotherapist was very difficult for 47% of the children compared to 58% of the adolescents and adults. Finding a physiotherapist trained in CP rehabilitation was reported as very difficult for 61% of children and adolescents and 66% of adults. Physiotherapy was provided in a private outpatient practice for 27% in adolescents, 41% in young adults and 57% in adults over 25 years. Regular communication between health professionals was less common in adults compared to adolescents, indicating a sharp decline in access to multiprofessional services. Generally, the access to and satisfaction with physical therapy in adults is much lower compared to young people with CP (28).

Occupational therapists can play a key-role in the transition process of young people with disabilities fostering self-determination but their services are often limited to school-based services or are discontinued at age 18 (29). A study with young people age 18–22 with CP in Ireland compared those who were discharged from pediatric services and those remaining. Especially the rate in occupational therapy dropped to 37.5% post discharge compared to 80.0% pre-discharge (30).

In this study, 16.5% of participants reported to receive speech and language therapy, but 14.8% reported unmet needs. A study from France compared health service use in different age groups and found a frequency of speech and language therapy in 17% of the ambulatory 18–24 year old participants and only 6% in non-ambulatory participants (31).

In psychosocial services, we found high rates of unmet needs: support with filling applications (16.2%), social services (14.8%), information (16.3%), and counseling about sexuality (11.3%). In our study 19.2% received psychotherapy but 16.3% reported unmet needs in this area. Given the high prevalence of mental health problems in young people with CP the provision of services appears to be poor (32, 33). In sexual counseling we would like to address the issue, that participants may not be aware of such service or sexuality may be considered a private issue not relevant to medical care. A Dutch study with young adults with CP described high levels of sexual problems mostly related to the fact of having CP and many young people wanted more information; however, in 90% the issue had never been discussed during rehabilitation treatment (34).

Unmet needs in rehabilitation (16.5%) and access to self-help groups (15.2%) indicate barriers to services aiming at fostering independence and self-efficacy. The need for home nursing was comparatively low in our sample. A study with a sample with CP aged 4–27 years demonstrated a considerable decrease of specialized rehabilitation services in the young adults compared to children and low levels of formal respite services and support groups/youth clubs (35).

The often abrupt transition from mostly well-equipped centers for integrated multiprofessional healthcare available for children and adolescents to a much more fragmented care in adulthood appears counter-intuitive as health in people with CP decreases over time and more but not less healthcare is required to prevent especially chronic pain, fatigue and declining walking ability (36). A systematic review of observational studies showed transition-associated poor outcomes including housing instability, unemployment, difficulty forming relationships, increased hospital admission rates, and decreased use of rehabilitation services. Factors associated with improved outcomes included family participation, promotion of self-efficacy, and meeting the adult team before transition (7).

Moreover, as special healthcare needs were assessed using two different assessments in our study (YHC-SUN-SF, EACD) the overlap between corresponding responses to both different measures ranging between 80 and 98% for physical therapy and occupational therapy, providing even stronger evidence for the reliability of this finding.

*Satisfaction with healthcare* was moderate to good and almost consistently higher for the sample of young adults with CP as compared with data of young adults sampled from the general population. Likewise, in a recent study from our research group, we also found better quality of life for the environmental domain in young adults with CP as compared to reference values for young adults from a sample of the general population (37). The Transition Collaborative Group in UK used a different approach measuring satisfaction with healthcare: the Mind the Gap scale measures the difference or “gap” between a young person’s ideal service and the service they have received. The results show decreasing levels of satisfaction with age; appropriate parental involvement was significantly associated with higher satisfaction in all age groups (38).

In this study, satisfaction with healthcare services was similar to that of the general population sample of the same age. Several factors may play a role: the recruitment in the general population survey did not exclude any other chronic health conditions. We also speculate that young adults with less exposure and interaction with healthcare providers may be more critical while the young adults with CP are likely to experience long lasting positive relations with at least some professionals in their healthcare. Higher involvement may lead to a higher appreciation of the services received.

*Associations of healthcare satisfaction* with severity of the condition and functional abilities were not substantial. A large French study including 354 children, 145 adolescents and 511 adults with CP measured satisfaction with motor rehabilitation services identified significant factors that decreased satisfaction: being an adolescent, higher levels of motor impairment, frequent pain, receiving physical therapy in private practice and poor access to a physiotherapist with specific CP training (39). Because the outcome was limited to satisfaction with a specific service whereas our study probed for satisfaction with healthcare in general the different findings are difficult to interpret.

Also, satisfaction with healthcare was not associated with utilization of healthcare services or (unmet) healthcare needs. Thus, unmet needs and satisfaction with healthcare seem to be unrelated constructs. In the Dutch PERRIN-PiP study a similar lack of association between satisfaction with another outcome was noted: their study revealed a dissociation between participation accomplishment and satisfaction with participation among adolescents with CP. The authors recommend not only to focus on accomplishment but also, if not mainly, on satisfaction (40).

### Strengths and limitations

Our study reveals some *strong features*: We assessed a large sample of young adults with CP from different regional contexts and countries of Europe. In addition, a large proportion of this sample included patients that were longitudinally assessed throughout their childhood and adolescence (41–43). Moreover, we managed to collect reference data from young adults with various chronic health conditions, based on population-wide surveys in the respective countries. Finally, most of the constructs used in our analyses were assessed using different indicators, e.g., for (unmet) special healthcare needs (YHC-SUN-SF, EAEQ), for satisfaction with care (YHC-SUN-SF generic single item vs. total scale score) or severity of condition (e.g., GMFCS, BFMF).

One *limitation* of the study is that all results are based on self- or proxy-reported data only, thus we did not include any objective assessment for utilization of healthcare services. Thus, the validity of the subjective responses about individual behavior remains questionable. Furthermore, reference values were not eligible for all countries included and for some we collected only small samples. However, also the results from the countries with smaller sample sizes confirm the evidence provided by population-based surveys, indicating quite similar findings. Beside of this, we also missed to include CP and GP samples from Eastern European countries, thus our study is biased to Western Europe.

## Conclusion

Young adults with CP reports of unmet healthcare needs varied largely but showed substantial deficits in some aspects. During the transition period there must be sustained support and access to condition specific treatments such as physical therapy, occupational and speech and language therapy, psychosocial services and counseling and care coordination to allow growth of self-determination and autonomy.

Unmet needs do not have an impact on the satisfaction with healthcare these patients currently receive and we conclude that these are two different concepts and separate indicators to evaluate the quality of healthcare services delivery.

## Data availability statement

The original contributions presented in the study are included in the article/supplementary material, further inquiries can be directed to the corresponding authors.

## Ethics statement

The studies involving humans were approved by Germany: Ethikkommission der Universität zu Lübeck (AZ 18-172). Italy: 352 Comitato Etico Lazio 1c/o A.O. San Camillo Forlanini (2143/CE Lazio 1). Sweden: Regional Ethical 353 Review Board in Göteborg. The studies were conducted in accordance with the local legislation and institutional requirements. The participants provided their written informed consent to participate in this study.

## Author contributions

HoM: Methodology, Writing – original draft. JA: Conceptualization, Investigation, Methodology, Writing – review and editing. CA: Conceptualization, Funding acquisition, Investigation, Methodology, Project administration, Resources, Supervision, Writing – review and editing. CC: Data curation, Investigation, Writing – review and editing. JF: Conceptualization, Investigation, Methodology, Project administration, Writing – review and editing. KH: Conceptualization, Investigation, Methodology, Writing – review and editing. MM: Conceptualization, Investigation, Methodology, Writing – review and editing. HeM: Conceptualization, Data curation, Investigation, Methodology, – review and editing. MR: Conceptualization, Investigation, Methodology, Writing – review and editing. SS: Conceptualization, Funding acquisition, Investigation, Methodology, Project administration, Resources, Supervision, Writing – review and editing. UT: Conceptualization, Funding acquisition, Investigation, Methodology, Project administration, Resources, Supervision, Writing – original draft.
